# Reducing Plastic Pollution by Recovery and Recycling: Evidence from a “Blue Economy” Project Impacting Policy-Making in Italy

**DOI:** 10.3390/ijerph20085604

**Published:** 2023-04-21

**Authors:** Lorenzo Vassallo, Andrea Appolloni, Chiara Fantauzzi, Rocco Frondizi

**Affiliations:** 1 Fondazione Angelo Vassallo Sindaco Pescatore, 00133 Roma, Italy; 2 Department of Management and Law, University of Rome Tor Vergata, 00133 Rome, Italy

**Keywords:** blue economy, circular economy, collaborative economy, solving plastic pollution

## Abstract

This paper recognizes the relevance of the Seabed Cleaning Project, created by the Angelo Vassallo Sindaco Pescatore Foundation in 2010, and presents it as a tool to achieve benefits in environmental, social, and economic terms, in line with the innovative framework of the Blue Economy. The project suggests a practical, feasible, and scalable solution to plastic pollution that can be obtained thanks to the activity performed by fishermen in cooperation with the municipality and local community (in a multi-level cooperation perspective). The data show a significant reduction of plastic on the seafloor thanks to the project, but further research is needed in order to collect further positive outcomes from different applications of the project. In 2022, the “Salva Mare” Law was approved in the Senate, extending the good practices proposed by the Foundation nationwide and showing how small gestures and concrete actions can provide significant improvements in pursuit of a healthier, safer, greener, and smarter city for the future.

## 1. Introduction

Focusing on the world ocean, this paper shows how plastic pollution represents a concrete threat to it and how multi-level cooperation is needed to solve it and ensure mutual benefits for all [1].

In a context where the world ocean collects up to 12.7 million tons of plastic every year from land [2], the most insidious problem is represented by micro-plastics, which are able to enter the food chain and damage the organism that ingested them [3], but also by the possibility for plastic to inhibit the ocean carbon and nutrient cycle, change the habitat, increase ecotoxicity, and modify societal behaviors [4].

The Blue Economy is a new way of developing the capitalistic society we live in, boosting sustainability in all its aspects [5] and characterized by vast possibilities for generating better food, cleaner energy, and healthier tourism [6].

By taking into consideration the possibilities of a healthy ocean developed by the Blue Economy, it is clear that humans need to act to solve plastic pollution. In line with these evidence, this research has the aim of presenting a socially innovative solution to plastic pollution since the theme is well studied but few have presented a feasible solution that is easily scalable. The study will be focused on the Seabed Cleaning Project, a practical solution developed by the Angelo Vassallo Sindaco Pescatore Foundation to reduce anthropic pollution on the seafloor and to stop river pollution by informing citizens and cleaning up the waterways. In this sense, it will be possible to coordinate the efforts of citizens and municipalities, enabling greater cooperation and mutual understanding among all the stakeholders focused on the same goal.

The research aims at answering the following questions: Has the Seabed Cleaning Project had a real impact on local plastic pollution? How will this project sustain the values of the Blue Economy? What are the possibilities for scaling it up?

To answer these questions, this paper will be structured as follows: after a review of the literature on the current state of the oceans, the new paradigm of the Blue Economy will be presented. The following section is dedicated to the project, looking for linkages with the Blue Economy approach: future perspectives will be developed on the basis of the previous performance of the project to hypothesize the related possible outcome if applied in other locations; results will be presented qualitatively.

The novelty of the study can be associated with its aim: it wants to present a practical application of the pioneering framework of the Blue Economy, demonstrating the relevance of cooperative approaches (among different actors within society) in order to achieve environmental, social, and economic benefits for all. Furthermore, differently from previous research, this paper focuses attention on the critical relationship between waste, people, and the decision to recycle or not, describing a project whose implementation has led to the approval of a new law in terms of marine waste management. With the Seabed Cleaning Project, it will be possible to create a drastic change in citizens’ mentality while supporting the efforts of the Public Administration in restoring the natural environment. The originality of such a project regards the local context where it has been applied, the Mediterranean, which is seen as a hub for wildlife.

Limitations regard the variable used to summarize data; it is expressed in kg, with the risk of underestimating or overestimating the impact generated by the project (depending on the weight of collected material). Furthermore, results turn out to be exclusively focused on the environmental aspect; currently, there is no recognized evidence for what concerns social and economic benefits.

## 2. Theoretical Framework

The theoretical framework of this study is composed of two main pillars: the first is aimed at describing the current ocean state and showing how plastic pollution is becoming a serious threat that must be faced through innovative approaches, while the second is dedicated to the concept of the Blue Economy, presented as a new capitalistic economy focused on respect for oceans and aimed to ensure benefits for all through a multi-level cooperation perspective.

### 2.1. The Current Ocean State

Every day, 730 tons of plastic waste enter the Mediterranean Sea, making it one of the most plastic-polluted areas in the world [7]. It is estimated that there are between 873 and 2576 tons of plastic fragments just floating in the Mediterranean waters [8]. Furthermore, it is estimated that, on a global scale, 73% of the plastic present in aquatic habitats is non-degradable [9]. This represents a fundamental risk for all living creatures, especially in a location that is considered a hub for wildlife, such as the Mediterranean. Researchers suppose that this area hosts between 4% and 18% of marine species [10] and 534 species of birds that migrate to and live in this hotspot. This richness needs to be protected by preserving their habitat and food chain.

Unfortunately, the study conducted by Pedrotti et al. [11] shows how micro-plastics are present throughout the Mediterranean basin, but especially in the first kilometer from the coast (from 28,000 to 578,000 Km^2^ fragments with a concentration of 56% above 100,000 fragments Km^2^) and the tenth kilometer onward. According to the same study, up to 40% of floating materials remain close to the coast for at least 30 years. This produces an accumulation of micro-plastics in the coastal area. Most of the materials, harmful to the ecosystem and of anthropogenic origin, come from rivers [12] in a larger form, which is then subjected to fragmentation with the sun and collisions and potentially becomes micro-plastics. The Mediterranean Sea shows a similar density and frequency of floating plastic concerning the five subtropical ocean gyres, but with a major difference regarding the larger size of fragments. It has been demonstrated that the entire EEZ of the Italian Peninsula is a section of great risk for the ingestion of micro-plastics by marine fauna [7].

The strong presence of plastic and other solid waste in the Mediterranean Sea creates great damage to the biological and economic systems. Approximately 641 million euros are lost every year due to plastic pollution in the area, especially in two of the major drivers of economic development in the basin: tourism and fishing. Those two industries are major sources of income for the region and suffer dramatically when plastic pollution increases.

Several are the proposed remedies to plastic pollution; among the most common is recycling. For this reason, some studies have analyzed this topic from various perspectives [13], but few have studied the relationship between the waste, the person, and the decision to recycle or not. In this sense, the Seabed Cleaning Project could be considered unique since its application has led to the approval of a new law in marine waste management.

### 2.2. The Blue Economy Paradigm as the Solution

It was reported that humans are irrational when recycling, especially if the object is distorted. This major problem is part of the reason why only a minor part of all material is recycled. For this reason, the idea of recycling needs to be strictly connected with the idea of a Circular Economy. At first sight, the two seem interconnected, but if the public is not aware of the various implications, this may cause great material losses. For these reasons, it is important to design a system where the producers of waste and waste itself can be managed in a coordinated framework. This idea was represented in the concept of Circular Economy. Currently, such a concept is being pushed by large corporations that are working toward improving their relationships with the Government, the Public, and the Environment. This strategy resulted in being anonymous, connected, political, uncertain, and based on different values [14]. The term Circular Economy counts hundreds of possible definitions, making it a complex idea and creating a concept similar to an umbrella term for political and business narrative [15]. For this reason, we have decided to consider this term as defined by the European Parliament, according to which “the Circular Economy is a model of production and consumption, which involves sharing, leasing, reusing, repairing, refurbishing and recycling existing materials and products as long as possible. In this way, the life cycle of products is extended. In practice, it implies reducing waste to a minimum. When a product reaches the end of its life, its materials are kept within the economy wherever possible. These can be productively used again and again, thereby creating further value”. This explanation was chosen because it represents the core of the concept while acknowledging that products inside the Circular Economy will reach a point where they will not be usable anymore. This consideration is at the core of new development strategies such as the Blue Economy, which can better retrieve and reuse materials by referring to oceans. Indeed, if the Circular Economy can be seen as an economic model oriented towards the elimination of waste and the efficient use of resources, the Blue Economy aims to foster growth and development without compromising the oceans’ environmental sustainability and coastal areas [16].

The Blue Economy is intended as a new capitalistic economy based on the ocean and focusing on quality rather than cheap prices. Existing literature on the new concept of Blue Economy is not very wide, mainly undertaken in order to individuate an appropriate definition for it, retracing the minimum criteria that can be considered necessary for a Blue Economy [6]. Nevertheless, there is ambiguity around what the Blue Economy is, what it encapsulates, and its practices [17]. As stated by Voyer et al. [18], such a topic is conceptualized in different ways among countries and actors. In this sense, the Blue Economy could be associated with the marine economy [19], even if with several variations for what concerns the focus on equity and ecological sustainability, collaborations with different actors related to maritime industries and sea-related activities [20], and governance and policy aspects [21].

Indeed, the Blue Economy represents a recent concept, originating from the United Nations Conference on Sustainable Development held in Rio de Janeiro in 2012; it is built on the idea that the traditional separation between socio-economic development and environmental degradation should be overcome by integrating conservation and sustainability in the management of the maritime domain.

The United Nations (UN) has played a pivotal role in establishing the term “Blue Economy” and its related principles, looking at the Blue Economy as a parallel paradigm to the Green Economy, with the aim to improve human beings and social equity while managing ecological scarcities and environmental risks [22].

On the other hand, the European Union recognized the Blue Economy as an innovative paradigm for regional economic growth, able to reduce the negative environmental impacts of maritime activities such as the emissions of pollutants and the discharge of noxious substances [23].

Furthermore, of the main definitions to take into account, there is that from The Economist, according to which “a sustainable ocean economy emerges when economic activity is in balance with the long term capacity of ocean ecosystems to support this activity and remain resilient and healthy”, while in the Rio + 20 Green Economy Initiative it is presented as a concept able to generate positive outcomes in terms of “improved human wellbeing and social equity, while significantly reducing environmental risks and ecological scarcities, endorsing low carbon, resource efficiency, and social inclusion”.

According to the World Bank, a “Blue Economy is a marine-based economic development that leads to improved human wellbeing and social equity, while significantly reducing risks and ecological scarcities”.

Other terms such as “Ocean Economy” or “Coastal Economy” were used by the U.S. National Oceanic and Atmospheric Administration (NOAA) to indicate all the activities related to the coastal and water environments and to specific economic sectors.

On the other side, Pauli [24] presented the Blue Economy as a model based on technological innovation to supply products at low cost, promote local job creation, and be respectful of the environment while being competitive in the market.

Research on the Blue Economy turns out to be disparate and multi-faceted, characterized by different perspectives: if Winder and Le Heron [25] called for a critical engagement with the Blue Economy, Bear [26] clarified the ontological separation between land and sea, while others conceptualized the Blue Economy as a project related to new and particular ways of governing [27,28].

To bring things to an end, an overall definition is provided by Smith-Godfrey [6], according to which the Blue Economy is “the sustainable industrialization of the oceans to the benefits of all”. This definition shows that the concept of the Blue Economy derives from the Green Economy, looking for balance between the three different spheres (economic, environmental, and social); it involves the economic activity of a specific area operating in a non-traditional environment represented by the oceans. Furthermore, according to such a definition, the aim of the Blue Economy is to ensure equity and wellbeing for both humankind and the environment through a holistic and all-inclusive approach.

It is an innovative way of developing that can sustain economic growth at all levels and across all nations, regardless of their proximity to the sea. This expansion inland is due to the vast possibilities that the ocean is capable of giving to human development, including providing food, oxygen, and livelihoods. Indeed, the world ocean is capable of providing clean and reliable energy [29] or food sources that can be transported inland. Furthermore, the ocean is vastly unknown; the U.S. National Oceanic and Atmospheric Administration (NOAA) estimates that 80% of it is undiscovered, making it an immense field of study for possible breakthroughs in various fields. Those studies can provide our society with new knowledge in medicine [30], Ocean Chemistry, and Ocean Engineering, and innovations will be developed regardless of their geographical location, giving people all over the world the chance to experience the Blue Economy.

Unfortunately, the entire ecosystem is threatened by human activities. It is possible to identify five different types of activities related to the oceans [6], namely:

- Harvesting of living resources: the oceans provide food security for humans and animals, fostering the development of the aquaculture and mariculture industries;
- Extraction of non-living resources: what concerns seabed mining and shallow-water mining for minerals and metals;
- Generation of new resources: the oceans offer water and are able to generate alternative and renewable energy, facing the increasing demand for energy, a lower carbon footprint, fresh water;
- Trade of resources: including the transportation of resources and the service of transport, linked to the shipping industry and the activity performed by ports and coastal infrastructures;
- Resource health: looking at ocean surveillance and monitoring, coastal governance, and ocean management.

The ocean is becoming warmer and more acidic due to climate change, and all living beings will sooner or later experience a distortion in their lives. The effect will be felt by marine creatures such as fish and corals as well as humans. The Anthropocene is not stopping, and something must change to preserve the planet. It is necessary to find a balance between respect for sustainable economic values and the traditional economic activity performed by communities.

It is interesting to note that, despite the relevance of the Blue Economy within society, very little is known about its interactions or interconnections with the total environment, which are often conflictual and characterized by the growth/development and protection of ocean resources [31].

The Blue Economy is associated with certain complexity and subjected to a multilayer, multidimensional regulatory framework, developed thanks to the United Nations Conventions on the Law of the Sea, ratified by countries all over the world.

Silver et al. [32] and Voyer et al. [18] performed a different kind of analysis, proposing alternative framings such as “ocean as natural capital”, “oceans as livelihoods”, “ocean as good business”, and “ocean as a driver for innovation”, while Winder and Le Heron [25] tried to identify potential connections in order to develop innovative behaviors able to ensure sustainable collective and individual benefits from the oceans.

Lee et al. [33] linked the Blue Economy to the UN Sustainable Development Goals and identified precise objectives for the Blue Economy, such as underwater life, land ecosystems, peace, justice, and stable institutions and alliances to achieve the objectives.

Sustainable development requires agreement and cooperation between several actors, including those who live and work in coastal areas [34], fostering a “bottom-up” approach able to ensure effective sustainable processes and the respect of social justice principles. Indeed, it is important to generate synergies between different sectors, combining a multi-level perspective with a multi-stage concept, and recognizing individual initiatives as crucial factors to obtain a more sustainable approach [35].

Alongside climate change, there is plastic pollution as a source of physical danger to creatures. Plastic is killing wildlife across the globe [36,37], and this poses a threat to the entire concept of the Blue Economy. For this reason, the reduction of plastic means supporting this new style of sustainable development.

Furthermore, the paradigm of the Blue Economy helps to achieve specific Sustainable Development Goals, such as SDG14, which is completely based on it, but also SDG1, which ensures the eradication of poverty; SDG2, which ensures the eradication of hunger; and SDG10, which ensures the reduction of inequalities within and among countries [38,39,40]. Moreover, it is in this sense that the sustainable marine economy has become a priority all over the world [41], with the aim to maintain the health of the marine ecological environment and achieve relevant results in terms of the Blue Economy [42,43]. According to Choudhary et al. [44], today the Blue Economy plays a crucial role in sustainable global economic development, and for this reason it has been reorganized by human society, becoming a research hotspot [45].

As shown above, the only way to solve plastic pollution in the ocean is through a multi-stakeholder approach, from national governments to local communities, and different perspectives have been developed. Crona et al. proposed a framework according to which collaboration in the marine sector will ensure sustainability and equity in the use of marine resources [46], while, according to Cziesielski et al., the development of blue natural capital will maintain the sustainable development of marine ecosystems [47], and the growth of blue capital value can be seen as an indicator of the efficient use of marine resources, ecological environmental protection, and coordinated socioeconomic development [48].

It is important to highlight that the concept of a Blue Economy could generate different conflicts of interest, especially in what concerns economic growth and development on the one hand and safeguarding and protecting ocean resources on the other [49]. Consequently, the aim of the Blue Economy model should be to transfer resources from scarcity to abundance and try to solve the causes of environmental problems [50] by individuating solutions able to take advantage of all available opportunities and prevent the threats characterized by the external environment.

At the same time, as stated by Liang et al. [38], most of the Blue Economy development research appears independent, with little cooperation among different institutions—a sort of cooperation that is needed since the Blue Economy depends on the growth of different industries and activities [51,52,53].

Consequently, it is necessary to promote multidisciplinary cross-fertilization, allowing the development of different interrelated sectors, such as marine aquaculture, marine ship engineering and transportation, marine energy exploitation, marine tourism, the sea salt industry, etc. [38].

The Angelo Vassallo Sindaco Pescatore Foundation has developed a special project aimed at creating strong synergies between various stakeholders. Only if the civil society is motivated to protect the environment will the political agenda put this theme at the core of its initiatives, and in this sense, it is thanks to the Seabed Cleaning Project that a new law in marine waste management has been endorsed.

## 3. Methodology

As already stated, this paper has the objective of recognizing the relevance of the Seabed Cleaning Project, which can be seen as a tool to achieve social responsibility goals. In this sense, after the analysis of the outcomes related to the project, an estimation of its potential overall benefits is presented.

The theoretical framework of the study is composed of two main sections: the first one describes the current ocean state in terms of plastic pollution, while the other one introduces the new concept of the Blue Economy, able to ensure environmental benefits and economic development.

The practical section is dedicated to the project itself.

The data collection process was performed using two different methods. First of all, data were collected directly on the field through interviews with fishermen, who can be considered the main players engaged in the project. Through such a method, the researchers had the opportunity to interact with the main actors, generating new sources of information that are not possible to identify only through data. Furthermore, quantitative data were collected by the operators of the recycling facility during their normal routine and communicated weekly, allowing a more precise reconstruction of undelivered data. The information was reported in kilograms due to the ease that this measurement method allows the workers, since other methodologies such as volume calculation were less applicable in this context due to the few minutes the workers had to perform this task. Data were deeply reliant on the number of fishing boats going to sea; this made data more volatile if one or more boats decided to remain docked.

Finally, data were analyzed in a quantitative and qualitative way, demonstrating the value of the Blue Economy as a valid alternative for the world, able to generate environmental, social, and economic benefits. In terms of future perspectives, it is possible to think about further application of the project; for instance, it could be applied in different locations and used to foster awareness and education on sustainable development topics at school.

The originality of the study consists of the description of a longstanding activity that has led to the introduction of a new law and that can be easily scalable worldwide, while limitations regard the lack of evidence for social and economic benefits.

## 4. The Seabed Cleaning Project

The Seabed Cleaning Project was proposed and implemented by the Angelo Vassallo Sindaco Pescatore Foundation with the aim of ensuring continuity to the efforts made by Angelo Vassallo, the fisherman mayor of Pollica (Salerno, Italy), who was killed on 5 September 2010 in an assassination attempt allegedly of camorristic origin.

Today, we can consider Angelo Vassallo as an innovator, certainly as one of the pioneers of certain practices of social responsibility, who had the goal of protecting our environment, especially the sea, while ensuring economic development, and who strongly believed in the power of small gestures and concrete actions in order to achieve significant improvements.

Angelo’s idea stems from the observation of the worrying situation characterizing the Mediterranean Sea, where 731 tons of garbage are thrown away every year, and aims to create a synergy between different parties, such as fishermen, the municipality, and the local community of reference, with the intention of recognizing and enhancing the activity of cleaning the seabed freely carried out by the fishermen of Acciaroli and Pollica. The Mediterranean Sea is a closed one, into which as many as 69 rivers flow, where water recycling occurs every 100 years through the Strait of Gibraltar, and on whose shores approximately 150 million people live (reaching 450 million during the summer period). In addition, in many places in Italy there are no adequate purification plants, which is why Italy is under infringement proceedings by the European Union, risking a fine of millions of euros. In this regard, each country should aim to increase marine protected areas, with the goal of achieving 30 percent coverage of the ocean’s surface area by 2030. This is where the work of the Foundation comes in, looking at the marine areas not only as safeguard zones but also as zones of potential economic development.

In this regard, practically speaking, the project consists of sorting and stowing all the material (from plastic to glass, from aluminum to iron, etc.) that is retained by fishermen’s nets in their trawling activities; this is extremely dangerous material, capable of altering the state of the seabed, doing enormous damage to marine ecosystems, and generating a very heavy negative impact on the habitat. These are devastating effects in terms of pollution, survival of marine fauna, and risk of entering the human food chain.

Involuntarily, every day, each individual fishing vessel recovers between 30 and 50 kg of waste, which is dumped at the port and then picked up and sorted at the ecological island. Considering the activity of six different fishing vessels, about 300 kg of material is potentially recovered daily, which multiplied by 200 working days becomes as much as 60 tons per year.

However, all of this remains useless without the legitimization of the project and the permission from the port authority to collect the plastic at sea and then bring it to port. In fact, in Italy, when the project was launched, the law considered the material collected at sea as “special waste”, requiring specific procedures regarding its management and therefore its disposal.

In spite of this, in November 2010 and thanks to the contributions of different categories of stakeholders, such a project began, involving three different phases:

1. The first phase concerns the collection of the waste: during the course of their normal working activities, the fishermen collect the material by separating it from the catch; this is an activity that does not negatively impact the efficiency of their work since it has to be performed anyway. At the end of the working day, when the fishing vessels return to port, the recovered material is collected inside plastic containers that remain on the vessels until they are full;
2. The second phase begins once the plastic containers have been filled. These are deposited by the fishermen near the harbor so that they can be easily collected by the waste handlers;
3. The third and final phase involves the actual handling of the waste: it is sorted and washed at recycling facilities.

This is a cultural project capable of changing the conception of fishermen, who no longer operate as predators of the sea but rather protect and cultivate it by going out and picking up material that would otherwise never be picked up, lying on seabed ranging from 50 to 600 m deep.

**Figure 1:**
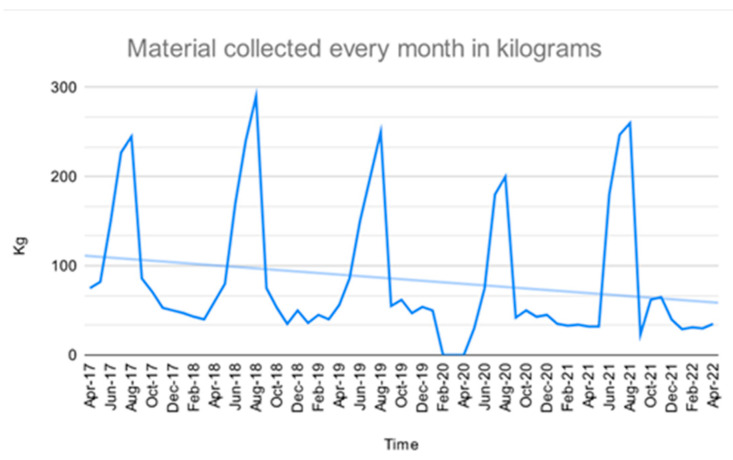
The amount of collected material from April 2017 to April 2022 (in kg).

In the first years of the project, the Foundation estimated an average daily amount of 30 kg of plastic collected on the seafloor. Currently, the weight of materials is less than in previous years, as shown in Figure 1.

The graphical representation of the data shows a correlation between the months and the amount of materials collected in kilograms. As shown, the amount of plastic pollution collected during each month varies dramatically between the summer months and the winter season. The analysis shows an average downward trend in collected plastic, especially in the last few years. These data suggest a possible plateau where the amount of pollution constantly emitted into the sea is collected. The graph represents two major trends: summer and winter.

In the summer months, the fishermen recollect more material than in the rest of the year, starting in late May and reaching the first week of September. This is strictly correlated with the increased number of tourists in the area. Indeed, as the summer months became more crowded, the fishermen collected more plastic. By analyzing the recollected material, the reason is clear. A multitude of plastic bags filled with leftovers, plastic plates, consumed water bottles, and other items related to mealtimes. This is not to be intended as poor performance of the collection facility; it is attributed to tourist actions. Sometimes, during the interviews with fishermen, it was reported that the yacht tourists were the ones who polluted the most. It is reported that this category of visitors disposes improperly of their garbage by simply outboarding it. This is a trend reported since the beginning of the project and represents one of the major drivers of sea pollution during the summer season. The authorities are not able to stop this phenomenon due to their inability to catch the transgressors while the action is occurring.

After August, the graph has a sudden and sharp decline, which is caused by the period of biological rest imposed by law. Italian regulators have divided the peninsula into several coastal regions to better manage the 30-day biological rest period. In Acciaroli, this period starts from the first week of September to the first week of October, which explains why the sudden decline in plastic collection was noted.

In the autumn and winter seasons, the plastic collected may be wildly varied. If the meteorological condition is adverse, the amount of plastic collected the following days is greater than the average. This is because the river system is not controlled by the municipality and, in some cases, is used as a dumping site. With heavy rains, water flow increases and recollects more material near the banks, making it reach the sea. The increase in plastic may also be explained by stronger sea currents and waves during bad weather. The tumultuous sea pushes seabed pollution across the coast, making cleaner areas polluted again. These two outcomes of storms passing by make every fishing boat collect some quantity of plastic.

As mentioned, the fishermen were carrying out this activity for free and without asking for anything in return, but in order for them to be able to continue to do so, it was appropriate to provide incentives towards them and in support of the entire fishing sector, which has evidently been in crisis in recent years, partly due to the high cost of fuel.

Additionally, it was with the aim of legitimizing what was envisaged in the project that, on 26 January 2012, the Foundation itself filed a proposal for a European law for what concerns the cleaning of the Mediterranean seabed. The proposal consisted of granting economic reductions to the captains of fishing boats, going so far as to involve all municipalities that had the possibility of picking up waste at sea and then managing it; in fact, the municipalities called to adhere were those equipped with ecological islands and characterized by a separate collection of more than 50 percent. By providing economic rewards for municipalities in relation to the amount of material collected and sorted, the law is able to help the fishing sector in a concrete way.

In addition to the cleaning of the seabed, the project was also born with the intention of changing the rules and regulations regarding the biological closure, a completely useless practice if we consider that after a long period of closure, at the time of resumption of activities, the catch is excessive and consists of small fish, generating within a few days a reduction in the catch itself. In order to find a solution to the above problem, in 2016 the Foundation presented a proposal at the World Conference on Oceans, suggesting that the sea and oceans should be divided into many rectangles (based on different parameters to be taken into account), alternating rectangles where fishing is banned with rectangles where fishing is allowed.

Additionally, in 2016, the idea presented by the Angelo Vassallo Sindaco Pescatore Foundation was taken into consideration by the Apulia Region, which proposed a regional law concerning the cleaning of the seabed with the help of fishermen.

On 11 May 2022, the “Salva Mare” Law (the law that saves the sea), dedicated to Angelo Vassallo, was finally approved in the Senate, under which waste accidentally caught at sea is equated with waste from ships, favoring its collection and disposal by the fishermen themselves. Thanks to this new law, the good practices proposed by the Seabed Cleaning Project are now extended to all active fishing vessels nationwide, which will be able to deliver the waste recovered during fishing to the port collection facility. The related management costs will be covered through a specific component that will be included in the waste tax bill (“Tari”), with the Regulatory Authority for Energy Networks and Environment having the task of regulating the application methods.

The cleaning campaigns can be initiated both at the initiative of municipalities and at the initiative of promoting entities, which include the managing bodies of protected areas, various associations (environmentalists, sport fishermen, trade associations, etc.), as well as managers of bathing establishments, non-profit organizations, and other entities identified by the competent authority. In order to incentivize the adoption of the aforementioned practices by fishing vessels, reward measures are envisaged for the most virtuous.

The approval of a law on the cleanliness of the sea is now the best way to honor Angelo Vassallo, who dedicated his entire life to the defense of the environment and legality, and the best way to follow up on an activity despite the killing of its promoter.

Further recognition came through an official invitation to the Angelo Vassallo Foundation to participate in the 2022 United Nations Ocean Conference held in Lisbon, Portugal. At the conference, the Foundation presented the “*Salva Mare”* Law as a tool through which to achieve social responsibility goals.

## 5. Discussion

In a world that continues to produce plastic, this research has the aim of recognizing the relevance of the Seabed Cleaning Project, which is able to generate a positive impact on the local environment by reducing plastic pollution and boosting cooperation among different players.

The previous paragraph shows that, on the basis of data collected during the first year of implementation, it could be possible to recover up to 60 tons of plastic per year. However, beyond such quantitative results, the real practical implication related to the project is the possibility of changing fishermen’s conception: they no longer have to operate as predators of the sea, but they have to protect and cultivate it. Such change has to be verified over time, but for now we can state that the most concrete and important result achieved by the project is the approval of the “*Salva Mare*” Law, which will encourage fishermen to collect and dispose of plastic material, recognizing and attributing value to activities that before were performed involuntarily by the fishermen themselves. It is important to state that until plastic pollution stops entering the ocean, the project will not be able to completely restore the seabed to its pre-plastic state. To fight this situation, the Angelo Vassallo Sindaco Pescatore Foundation is promoting a new version of the project that aims at cleaning the seabed and rivers but also at promoting the topic of sustainability in schools in order to foster a new awareness and to push for a more sustainable future. With this implementation, the ambition pursued by the Seabed Cleaning Project is to reduce plastic consumption and pollution inland and near the coast.

The Blue Economy is a valid alternative for the world; it combines sustainability with a capitalistic mindset. Its basis is rooted in the positive relationship between profit and environmental protection and in the needed cooperation among different levels. This interaction must consider all stakeholders and work with a long-term perspective; unfortunately, this type of development is deeply reliant on ocean health. For this reason, economic development must be coordinated with environmental efforts to protect this global common. The project proposed is a way to protect the ocean from severe damages caused by humans, especially in terms of plastic recycling and community engagement. The fishermen interviewed report how important this project is for them; they feel part of a bigger picture and trusted to be the keepers of the sea. This sense of empowerment can push entire communities to make changes in their lifestyle and their relationship with the environment surrounding them. With this push toward a better future caused by the Seabed Cleaning Project, it is possible to shake up the way we perform in our economy. The project presented can deliver what has been promised at the various levels of commitment; the possibilities are vast, especially if it is taken into consideration with the new implementations developed by the Foundation. Possible participants are small to medium municipalities on the coast and cities with rivers or lakes inland. These two approaches are perceived as the best ways to reduce the amount of plastic in rivers and the ocean and also spread important knowledge on sustainability. It is this factor that makes seabed cleaning an attractive option. It is possible to scale its activity rapidly and start performing immediately. This ability was created over the years, and now it is possible thanks to the new Italian law “*Salva Mare*”, proposed by the Vassallo Foundation, dedicated to Angelo Vassallo, and approved in the Senate. With the legislative framework better prepared for this type of activity, the Vassallo Foundation is aiming at activating this project in several locations both inland and on the coast. This new law is a testimony to how important the concepts of social innovation and sustainability have become in defining (and hopefully improving) the future of our world.

## 6. Conclusions

This work is the summary of eleven years of activity aimed at restoring the sea, but it is also a way to attest to and honor the efforts performed by Angelo Vassallo and then by the Angelo Vassallo Sindaco Pescatore Foundation.

During this period, several major occurrences have demonstrated the importance of a common framework to address the main threats to human life: according to D’Adamo et al. [54], in order to achieve an ecological transition, a new social approach based on citizens’ involvement in decision-making processes is needed. Such an idea is at the basis of the Seabed Cleaning Project, which aims to reverse the course of action by looking at the Blue Economy as a valid alternative for the world. Indeed, it is clear that this project needs a strong cultural and political base to prosper; it needs to change human mindsets, and this article has the aim of pushing this message forward.

After a mainly theoretical background dedicated to the new concept of the Blue Economy, the Seabed Cleaning Project has been presented as an innovative practice where engaging citizens (especially fishermen) is able to rehabilitate the marine environment, ensuring benefits for the fishing sector and society.

The project asks for multi-level cooperation and can be easily scalable worldwide; these characteristics justify its originality and the choice of analyzing it. Data have been collected directly in the field in order to generate secondary sources of information and analyzed in a quantitative and qualitative way, but the high relevance of the topic described requires more detailed data to create a better analysis and to provide purposefully targeted solutions.

Furthermore, the methodology using kg neglects the impact that the project has in recollecting light materials (bottles, straws, pipes, bags, food) and overestimates when heavier items are recollected (washing machines, tires, metal). Other limitations regard the lack of evidence for social and economic benefits.

For this reason, it is important to consider this paper as a blueprint for further works related to the Seabed Cleaning Project and to think about measures able to retrace the social and economic benefits generated within society. The approval of a new law can be seen as the main social outcome achieved through the project, but related benefits should be measured and compared over time with the aim of understanding whether and how citizens’ mentality has changed. Furthermore, in terms of future perspectives, it will be interesting to test the efficacy of the project in different contexts by applying it to river cleaning and using it to improve citizens’ awareness, starting in schools. Through such applications, the project aims at recollecting more data from various sources to test its overall performance and elevate its value.

## Data Availability

The data presented in this study are available on request from the corresponding author.

## References

[1] Orste L., Ozola L., Lemberga K., Kills E., Adamsone-Fiskovica A., Grivins M., Tisenkopfs T. Conceptualizing and Identifying Social Innovation in Agri-Food Systems. https://graduate.aur.edu/sites/default/files/ORSTE_et%20al_Conceptualizing%20and%20Identifying%20Social%20Innovation%20in%20Agri-Food%20Systems.pdf.

[2] Haward M. (2018). Plastic pollution of the world’s seas and oceans as a contemporary challenge in ocean governance. Nat. Commun..

[3] Guzzetti E., Sureda A., Tejada S., Faggio C. (2018). Microplastic in marine organism: Environmental and toxicological effects. Environ. Toxicol. Pharmacol..

[4] MacLeod M., Arp H.P.H., Tekman M.B., Jahnke A. (2021). The global threat from plastic pollution. Science.

[5] Bennett N.J., Cisneros-Montemayor A.M., Blythe J., Silver J.J., Singh G., Andrews N., Calò A., Christie P., Di Franco A., Finkbeiner E.M. (2019). Towards a sustainable and equitable blue economy. Nat. Sustain..

[6] Smith-Godfrey S. (2016). Defining the blue economy. Marit. Aff. J. Natl. Marit. Found. India.

[7] Compa M., Alomar C., Wilcox C., van Sebille E., Lebreton L., Hardesty B.D., Deudero S. (2019). Risk assessment of plastic pollution on marine diversity in the Mediterranean Sea. Sci. Total. Environ..

[8] Suaria G., Avio C.G., Mineo A., Lattin G.L., Magaldi M.G., Belmonte G., Moore C.J., Regoli F., Aliani S. (2016). The Medi-terranean Plastic Soup: Synthetic Polymers in Mediterra-Nean Surface Waters.

[9] Bergmann M., Tekman M.B., Gutow L. (2017). Sea change for plastic pollution. Nature.

[10] Bianchi C.N., Morri C. (2000). Marine Biodiversity of the Mediterranean Sea: Situation, Problems and Prospects for Future Research. Mar. Pollut. Bull..

[11] Pedrotti M.L., Petit S., Elineau A., Bruzaud S., Crebassa J.-C., Dumontet B., Martí E., Gorsky G., Cózar A. (2016). Changes in the Floating Plastic Pollution of the Mediterranean Sea in Relation to the Distance to Land. PLoS ONE.

[12] Allsopp M., Walters A., Santillo D., Johnston P. (2006). Plastic Debris in the World’s Oceans.

[13] Goodship V. (2017). Plastic recycling. Sci. Prog..

[14] Camacho-Otero J., Boks C., Pettersen I.N. (2018). Consumption in the Circular Economy: A Literature Review. Sustainability.

[15] Corvellec H., Stowell A.F., Johansson N. (2022). Critiques of the circular economy. J. Ind. Ecol..

[16] European Commission (2020). The EU Blue Economy Report.

[17] Garland M., Axon S., Graziano M., Morrissey J., Heidkamp C.P. (2019). The blue economy: Identifying geographic concepts and sensitivities. Geogr. Compass.

[18] Voyer M., Quirk G., McIlgorm A., Azmi K. (2018). Shades of blue: What do competing interpretations of the Blue Economy mean for oceans governance?. J. Environ. Policy Plan..

[19] Morrissey K. (2017). Economics of the marine-Modelling natural resources.

[20] Doloreux D. (2017). What is a maritime cluster?. Mar. Policy.

[21] Zukauskaite E., Trippl M., Plechero M. (2017). Institutional Thickness Revisited. Econ. Geogr..

[22] UN (2014). UN Blue Economy Concept Paper.

[23] European Commission (2017). Report on the Blue Growth Strategy. Towards More Sustainable Growth and Jobs in the Blue Economy 2017.

[24] Pauli G. (2010). The Blue Economy: 10 Years, 100 Innovations, 1000 Million Jobs.

[25] Winder G.M., Le Heron R. (2017). Assembling a blue economy moment? Geographic engagement with globalizing biologi-cal-economic relations in multi-use marine environments. Dialogues Hum. Geogr..

[26] Bear C. (2017). Assembling ocean life: More-than- human entanglements in the blue economy. Dialogues Hum. Geogr..

[27] Choi Y.R. (2017). The Blue Economy as governmentality and the making of new spatial rationalities. Dialogues Hum. Geogr..

[28] Germond B., Germond-Duret C. (2016). Ocean governance and maritime security in a placeful environment: The case of the European Union. Mar. Policy.

[29] Tollefson J. (2014). Blue energy. Nature.

[30] Santos J.D., Vitorino I., Reyes F., Vicente F., Lage O.M. (2020). From ocean to medicine: Pharmaceutical applications of metab-olites from marine bacteria. Antibiotics.

[31] Lee K.-H., Noh J., Lee J., Khim J.S. (2021). Blue economy and the total environment: Mapping the interface. Environ. Int..

[32] Silver J.J., Gray N.J., Campbell L.M., Fairbanks L.W., Gruby R.L. (2015). Blue Economy and Competing Discourses in International Oceans Governance. J. Environ. Dev..

[33] Lee K.-H., Noh J., Khim J.S. (2020). The Blue Economy and the United Nations’ sustainable development goals: Challenges and opportunities. Environ. Int..

[34] Johnson T.R., Hanes S.P. (2018). Considering Social Carrying Capacity in the Context of Sustainable Ecological Aquaculture.

[35] Kelly C., Ellis G., Flannery W. (2018). Exploring Transition PATHWAYS as an alternative Approach for the Integrated Management of Irish Estuaries and Coasts.

[36] Wilcox C., Puckridge M., A Schuyler Q., Townsend K., Hardesty B.D. (2018). A quantitative analysis linking sea turtle mortality and plastic debris ingestion. Sci. Rep..

[37] Wabnitz C., Nichols W.J. (2010). Plastic pollution: An ocean emergency. Mar. Turt. Newslette.

[38] Liang J., Yin Z., Yang J., Li Y., Xu M., Li J., Yang M., Niu L. (2022). Bibliometrics and visualization analysis of research in the field of sustainable development of the blue economy (2006–2021). Front. Mar. Sci..

[39] Hernández-Delgado E. (2015). The emerging threats of climate change on tropical coastal ecosystem services, public health, local economies and livelihood sustainability of small islands: Cumulative impacts and synergies. Mar. Pollut. Bull..

[40] Leslie H.M., Basurto X., Nenadovic M., Sievanen L., Cavanaugh K.C., Cota-Nieto J.J., Erisman B.E., Finkbeiner E., Hinojosa-Arango G., Moreno-Báez M. (2015). Operationalizing the social-ecological systems framework to assess sustainability. Proc. Natl. Acad. Sci. USA.

[41] Sarker S., Bhuyan A.H., Rahman M.M., Islam A., Hossain S., Basak S.C., Islam M.M. (2018). From science to action: Exploring the potentials of Blue Economy for enhancing economic sustainability in Bangladesh. Ocean Coast. Manag..

[42] Weiand L., Unger S., Rochette J., Müller A., Neumann B. (2021). Advancing Ocean Governance in Marine Regions Through Stakeholder Dialogue Processes. Front. Mar. Sci..

[43] Singh G.G., Cottrell R., Eddy T., Cisneros-Montemayor A.M. (2021). Governing the land-Sea interface to achieve sustainable coastal development. Front. Mar. Sci..

[44] Choudhary P., Subhash G.V., Khade M., Savant S., Musale A., Kumar G.R.K., Chelliah M.S., Dasgupta S. (2021). Empowering blue economy: From underrated ecosystem to sustainable industry. J. Environ. Manag..

[45] Suris-Regueiro J.C., Garza-Gil M.D., Varela-Lafuente M.M. (2013). Marine economy: A proposal for ots definition in the European union. Mar. Policy.

[46] Crona B., Wassénius E., Lillepold K., A Watson R., Selig E.R., Hicks C., Österblom H., Folke C., Jouffray J.-B., Blasiak R. (2021). Sharing the seas: A review and analysis of ocean sector interactions. Environ. Res. Lett..

[47] Cziesielski M.J., Duarte C.M., Aalismail N., Al-Hafedh Y., Anton A., Baalkhuyur F., Baker A.C., Balke T., Baums I.B., Berumen M. (2021). Investing in Blue Natural Capital to Secure a Future for the Red Sea Ecosystems. Front. Mar. Sci..

[48] Fondo E.N., Ogutu B. (2021). Sustainable crab fishery for Blue Economy in Kenya. Aquat. Ecosyst. Health Manag..

[49] Martínez-Vázquez R.M., Milán-García J., de Pablo Valenciano J. (2021). Challenges of the Blue Economy: Evidence and research trends. Environ. Sci. Eur..

[50] Kathijotes N. (2013). Keynote: Blue Economy—Environmental and Behavioural Aspects Towards Sustainable Coastal Development. Procedia-Soc. Behav. Sci..

[51] OECD (2016). The Ocean Economyin 2030.

[52] Karani P., Failler P. (2020). Comparative coastal and marine tourism, climate change, and the blue economy in African Large Marine Ecosystems. Environ. Dev..

[53] Voyer M., van Leeuwen J. (2019). Social license to operate in the Blue Economy. Resour. Policy.

[54] D’adamo I., Gastaldi M., Morone P., Rosa P., Sassanelli C., Settembre-Blundo D., Shen Y. (2021). Bioeconomy of Sustainability: Drivers, Opportunities and Policy Implications. Sustainability.

